# Individual Stress Burden and Mental Health in Health Care Workers during the COVID-19 Pandemic: Moderating and Mediating Effects of Resilience

**DOI:** 10.3390/ijerph19116545

**Published:** 2022-05-27

**Authors:** Jonas Schmuck, Nina Hiebel, Milena Kriegsmann-Rabe, Juliane Schneider, Julia-Katharina Matthias, Yesim Erim, Eva Morawa, Lucia Jerg-Bretzke, Petra Beschoner, Christian Albus, Kerstin Weidner, Lukas Radbruch, Eberhard Hauschildt, Franziska Geiser

**Affiliations:** 1 Department of Psychosomatic Medicine and Psychotherapy, Medical Faculty, University Hospital Bonn, 53127 Bonn, Germany; jonas.schmuck@uni-bonn.de (J.S.); info@psychotherapie-hiebel.de (N.H.); milena.rabe@ukbonn.de (M.K.-R.); juliane-nora.schneider@posteo.de (J.S.); franziska.geiser@ukbonn.de (F.G.); 2 Department of Psychosomatic Medicine and Psychotherapy, University Hospital of Erlangen, Friedrich-Alexander University Erlangen-Nürnberg (FAU), 91054 Erlangen, Germany; yesim.erim@uk-erlangen.de (Y.E.); eva.morawa@uk-erlangen.de (E.M.); 3 Department of Psychosomatic Medicine and Psychotherapy, Ulm University Medical Center, University Ulm, 89081 Ulm, Germany; lucia.bretzke@uni-ulm.de (L.J.-B.); petra.beschoner@uniklinik-ulm.de (P.B.); 4 Department of Psychosomatics and Psychotherapy, University Hospital Cologne, University of Cologne, 50923 Cologne, Germany; christian.albus@uk-koeln.de; 5 Department of Psychotherapy and Psychosomatic Medicine, Faculty of Medicine, Technische Universität Dresden, 01069 Dresden, Germany; kerstin.weidner@uniklinikum-dresden.de; 6 Department of Palliative Medicine, University Hospital Bonn, 53127 Bonn, Germany; lukas.radbruch@ukbonn.de; 7 Faculty of Protestant Theology, University of Bonn, 53113 Bonn, Germany; ehauschildt@uni-bonn.de

**Keywords:** resilience, demographics, stress, COVID-19, health care, mental health

## Abstract

**Introduction:** The COVID-19 pandemic generated a significant burden on the German health care system, affecting the mental health of health care workers (HCW) in particular. Resilience may serve as an essential protective factor for individuals’ well-being. **Objective:** Our objective was to identify demographic and work-related correlates of individual resilience and to investigate the association between pandemic-related stress, resilience and mental health using different resilience models. **Methods:** Our sample comprised 1034 German HCW in different medical professions who completed an online survey from 20 April to 1 July 2020. Resilience was assessed using the Resilience Scale-5 (RS-5). The pandemic-related self-reported stress burden was captured by a single item, while depression and anxiety symptoms were measured with the PHQ-2 and GAD-2, respectively. Additionally, various sociodemographic and work-related factors were assessed. **Results:** Overall, we found high levels of resilience in the sample compared to a German sample before the pandemic, which were significantly associated only with the older age of participants and having children in both univariate and multivariate analyses. Regarding mechanisms of resilience, moderation analysis revealed that low individual resilience and high pandemic-related stress burden independently contributed to both anxiety and depression symptoms while resilience additionally moderated the relationship between stress burden and anxiety symptoms. The link between self-reported stress burden and mental health symptoms was also partially mediated by individual resilience. **Conclusion:** Taken together, the findings based on the present sample during the COVID-19 pandemic suggest that resilience plays a central role in the mental health of healthcare workers and that resilience-building interventions should be expanded, especially with a focus on younger employees.

## 1. Introduction

The COVID-19 pandemic and subsequent measures to contain the transmission of the SARS-CoV-2 virus led to major disruptions of personal and professional life in 2020. Not surprisingly, the number of people with mental health issues worldwide increased substantially in comparison to the time before the pandemic [1,2]. Health care workers (HCW) in particular have been among those most affected by the pandemic. They were exposed to unknown risks while treating patients with inadequate protection, concerned about infecting close ones with COVID-19 and working long hours [3,4]. Similar to previous epidemics [5], studies revealed high prevalence numbers for mental symptoms among HCW during the first wave of the COVID-19 pandemic. Reviewing 65 studies from mostly Asian countries, Batra et al. (2020) showed that the pooled prevalence of anxiety and depression in HCW was 34% and 32%, respectively [6]. In Germany, Morawa et al. [7] established that the prevalence across different groups of HCW was around 20% for both depression symptoms and anxiety symptoms. The sample investigated here is derived from the same survey. Not only that these figures make evident that HCW are exposed to high psychological stress, research additionally suggests that pandemics can be potential traumatic experiences for individuals [8]. Again, HCW in particular were found to be at high risk of developing post-pandemic post-traumatic stress disorder (PTSD) [9]. Therefore, it is important to identify factors that improve mental health and well-being in the long term. A psychological concept that has been associated with a stable trajectory after stressful events and psychological health is resilience [10,11].

Resilience has been the subject of different definitions such as “the ability of adults who are exposed to an isolated and potentially highly disruptive event […] to maintain relatively stable, healthy levels of psychological and physical functioning” [10] or as “a personality characteristic that moderates the negative effects of stress and promotes adaption” [12]. Although common themes can be identified in all definitions [13], there is still debate about the concept itself. Early studies approached resilience from a trait-oriented perspective [12], assuming that resilience is stable and inherent in all individuals. However, recent research views resilience rather from an outcome- and process-oriented approach, focusing on dynamic and temporal features [14,15]. Within this perspective, stable personality traits are seen as resilience factors predicting a favorable outcome. Even though this approach has become more popular in recent years, to date there is still no consensus about the best way to measure resilience [14]. In particular, most of the currently available and validated instruments (e.g., Resilience scale (RS) [12] or Connor–Davidson Resilience Scale (CD-RISC) [16]) view resilience rather from a trait-oriented perspective, in which resilience is operationalized as a personally available resource or “inner strength” that helps to cope with crises. The RS is separated into the two factors, *personal competence* and *acceptance of self and life*, while the CD-RISC reveals five factors such as *personal competence* and *control*.

In a review of 60 studies, a relationship between higher resilience and better mental health outcomes in the general population was reported [11]. Similarly, resilience has been positively associated with mental well-being in HCW, both in nurses [17,18] and general practitioners [19]. During the COVID-19 pandemic, several studies revealed that among HCW, higher resilience was correlated with fewer anxiety [20,21,22] and depression symptoms [22,23]. Since resilience shows a strong positive correlation with well-being, identifying underlying demographic and work-related factors of HCW with high resilience may help to recognize vulnerable groups in the face of stressful events. Earlier research among Singaporean HCW reported that older age, greater work experience and being married predicted higher levels of resilience [24]. Gillespie et al. (2009) found that among Australian nurses, only work experience remained as a significant positive predictor of resilience in multiple regression analysis [25]. In contrast, findings by Mealer et al. (2012) indicated that highly resilient nurses have little professional experience and tend to be older at the same time [18]. The heterogeneity of findings for nurses is highlighted in a recent meta-analysis comprising 33 studies that found no clear association between resilience and demographic variables [26]. In physicians, demographic factors also did not have a clear association with resilience [27]. Further studies among HCW revealed that higher resilience scores were associated with higher age and differences among professional groups that disappeared when taking other demographic and occupational factors into account [28]. Among British HCW both demographic (female gender and older age) and job-related factors (working at least half-time) were associated with higher resilience scores [29]. Due to the heterogeneous findings with regard to the role of demographic and occupational variables and the lack of findings originating from Germany, there was a need to examine associations in a German sample of HCW.

In addition to demographic and work-related correlates, there is also interest in explaining possible mechanisms of resilience in the face of stressors that improve mental health outcomes. Three models have been proposed for the mechanisms by which a protective factor, i.e., resilience, modifies the relationship between risk exposure and outcome [30,31]: The first one is called the compensatory model, which postulates that both resilience and potential stressors have an independent, additive effect on the outcome. The second model, the protective model, suggests an interaction between resilience and stressor, such that resilience moderates the effects of the stressor on the outcome. The third model, the challenge model, postulates a curvilinear relationship between stressor and outcome with different outcomes at different stressor levels [30]. Few studies have explicitly tested the moderating and mediating effects of resilience on mental health outcomes in HCW so far. In a sample of Chinese medical students, resilience correlated negatively with mental health problems and, in line with the protective resilience model, attenuated the effects of negative life events on mental health resilience as a protective factor [32]. In a Spanish sample of health workers, resilience mediated the relationship between job strain and several mental health variables [33]. During the COVID-19 pandemic, Havnen et al. (2020) reported that resilience moderated the association between stress experienced during COVID-19 and anxiety and depression symptoms [34], consistent with the protective model. The association between stress and psychological symptoms was attenuated in high-resilient individuals compared to low-resilient individuals. Although the study was conducted among the general population, it further illustrates the protective effect of resilience against acute stress caused by the COVID-19 pandemic. Therefore, our study aimed at testing resilience models in a sample of German HCW during the COVID-19 pandemic as no study has yet done this. Following the line of analysis by Anyan and Hjemdal (2016) [35], we specifically investigated the compensatory and protective model among HCW. Similar to their work, we also added mediation models to our analyses, e.g., resilience as a possible mediator between stressor and health outcome.

Although we share the concerns described above regarding the vague and malleable notion of resilience, and the accompanying problems of operationalization, we see resilience as a resource-oriented concept with a potential that merits further research. Our aim is to explore whether the concept of resilience can contribute to a better understanding of the relation between experienced burden and mental health, more specifically in HCW under the stress of a pandemic crisis.

To sum up, we can put forward the following hypotheses for this research work. As it has been shown in several studies from different countries, our first hypothesis was that socio-demographic factors (especially older age) and professional-related factors are associated with resilience levels among German HCW. Secondly, we hypothesized that resilience will show independent negative correlations with mental health outcomes, such as depression and anxiety levels, and moderating and mediating effects in the relationship between the subjective stress burden caused by the COVID-19 pandemic and mental health outcomes. In particular, in high resilient HCW, the effect of subjective stress burden on mental health symptoms should be attenuated in comparison to low resilient HCW. Additionally, resilience should mediate the relationship between subjective stress burden and health outcomes as in the work of Anyan und Hjemdal (2016) [35].

## 2. Materials and Methods

### 2.1. Data Collection

The online survey was conducted between 20 April and 1 July 2020. The link was provided through online platforms or mailing lists for the staff of the university hospital of Bonn and further general hospitals as well as several professional associations. The background for the study presented here was a research project on resilience in religion and spirituality, which was extended to general resilience during the COVID-19 pandemic. Data from the VOICE survey were used, which came only from Bonn. The data evaluation of the VOICE survey was performed as part of the collaborative research project egePan Unimed within the newly set-up German Network University Medicine. The aim of egePan Unimed is to examine and coordinate management concepts of the pandemic in Germany and internationally, to evaluate their practicability using scientific methods and to manage them within a framework plan. Superior aims include an adequate control of resources within a region in order to avoid an inefficient occupancy and intensive care supply in an inpatient setting and case management for both hospitalized and non-hospitalized patients.

The study was approved by the Ethics Committee of the Medical Faculty of the Rheinische Friedrich Wilhelm University Bonn (reference number: 125_20). All respondents provided their online informed consent.

The 20 min survey in the German language could be accessed via the academic online survey tool SoSci Survey (www.soscisurvey.de). The survey was made up of 82 items. Inclusion criteria were a minimum age of 18 years, working in the health care sector in a clinical setting, residence/working place in Germany and sufficient German language skills.

### 2.2. Sample Characteristics

In total, 1034 health care professionals took part in the online survey. The majority worked in university hospitals (*n* = 659, 63.7%), and of these 90% were from the university hospital of Bonn. Less than one-third of the participants worked in other general hospitals (*n* = 317, 30.7%). The remaining 5.6% indicated working in other institutions such as doctor’s practices or medical care centers. Regarding the different medical profession groups, 24.9% (*n* = 257) of the total sample (*N* = 1034) reported to work as a physician, 31.9% (*n* = 330) as a nurse, 14.9% (*n* = 154) as pastoral worker or psychologist (in the following referred to persons with psychological/spiritual care education (PPE)), 8.7% (*n* = 90) as medical technical assistant (MTA), and 19.6% worked in departments such as administration or emergency services. The term MTA refers to allied medical staff such as laboratory or radiology or pharmaceutical-technical assistants who have undergone three years of professional training.

To provide a partial measure for the representativity of the sample, we can report the response rates for the university hospital of Bonn for physicians, nurses and MTA. The highest response rate was found for the MTA (26.9%), followed by physicians (20.7%), and nurses (14.8%). The gender proportion (females to males) for the three groups was 63.0%:37.0% (respondents) and 47.7%:52.3% (hospital) for physicians; 75.5%:24.5% (respondents) and 70.8%:29.2% (hospital) for nurses, and 90.0%:10.0% (respondents) and 91.9%:8.1% (hospital) for MTA. Over 93 percent of the HCW participated within the first month after the study was launched (20 April—20 May).

### 2.3. Measures

#### 2.3.1. Mental Health

Mental health symptoms were measured with the PHQ-4 (Patient Health Questionnaire) [36]. This ultrashort form (4 items) of the Patient Health Questionnaire (PHQ-D) is divided into two separate modules, which indicate levels of depression (PHQ-2) or generalized anxiety (GAD-2), respectively. Each module consists of two items ranging from 0 (“not at all“) to 3 (“nearly every day“). An aggregate sum score with scores between 0 and 6 was computed for each module with cut-off values ≥ 3 to identify probable cases of clinically relevant depression or anxiety levels. The psychometric characteristics of the PHQ-2 and GAD-2 are well established [36]. In the present sample, the validated German version received acceptable Cronbach’s alpha scores of 0.71 for PHQ-2 and 0.76 for GAD-2.

#### 2.3.2. Subjective Stress Burden Levels

The overall self-reported subjective stress burden level during the COVID-19 pandemic was assessed on a single item basis. Participants were asked, “How much burden have you felt due to the COVID-19 pandemic in the last 2 weeks?” The scale ranged from 0 “not at all” to 4 “very strong”. Higher scores therefore reflect a higher subjective stress burden due to the COVID-19 pandemic.

#### 2.3.3. Resilience

Due to the lack of a gold standard for the assessment of resilience [14], and the need for an economic and validated measurement in an online survey, we chose the Resilience Scale-5 (RS-5) [37], which is based on the Resilience Scale-25 [12] and represents a highly abbreviated version. As in the original version, resilience in the RS-5 is defined as a “positive personality characteristic that enhances individual adaptation” [12]. The RS-5 is based on a unidimensional structure, which is determined by the factor of *personal competence* and includes self-reliance, determination and mastery [12]. The short scale has already been successfully validated and it was found to have a high model fit, and excellent goodness-of-fit criteria have been reported [38]. Subjects are asked to answer a total of five items on a seven-point Likert scale ranging from 1 (“No, I disagree”) to 7 (“Yes, I completely agree”). A total sum score with scores between 5 and 35 is calculated with higher values representing a higher degree of individual resilience. The RS-5 scale achieved a good Cronbach’s alpha value of α = 0.82.

#### 2.3.4. Sociodemographic, Occupational, and COVID-19-Related Variables

The online questionnaire consisted of several sociodemographic, occupational and COVID-19-related characteristics. The following data were used in our study: age, gender, living alone (or not), having children (or not), profession, years of professional experience, employment status and having direct contact at work with COVID-19 infected patients or having contact with contaminated material during work. The two latter conditions were aggregated into the variable: Having contact with SARS-CoV-2. Response categories for all items are presented in Table 1.

Other variables from the online survey not included in this paper were questionnaires measuring working conditions and potential problems in the COVID-19 pandemic, posttraumatic stress disorder symptoms, work–family conflict, and further resources; these have been published in other papers.

### 2.4. Statistical Analysis

All data analyses were conducted with SPSS V. 27. Descriptive statistics (absolute and relative frequencies for categorical variables or mean and standard deviation for continuous variables, respectively) were calculated to describe the demographic and work-related characteristics, resilience, mental health (anxiety and depression) and the subjective stress burden levels for the whole sample. To further explore relationships between the most important variables of interest, a correlation matrix was calculated. Pearson’s correlation coefficients and, in the case of ordinal variables, Spearman’s correlation coefficients were provided. A level of significance of *p* < 0.05 (two-tailed) was predetermined in all analyses, except for the case of alpha error correction (then explicitly reported in the text).

In order to identify associations between resilience levels in the RS-5 and demographic and occupational variables, Welch’s *t*-tests or the univariate analysis of variance (Welch’s ANOVA) [39,40] were used. If the ANOVA test for variables with more than two groups was significant, Games–Howell post hoc tests were conducted to highlight significant differences between the individual levels of the variable. To identify the best predictors for the resilience levels, taking into account all demographic and occupational variables, the effects of all variables were assessed simultaneously using a general linear model (GLM). The GLM allows the inclusion of continuous and categorical predictors and was therefore chosen for the present data. The effect size was reported both for the univariate as well as multivariate tests (Cohen’s d or η^2^_*p*_, respectively).

To evaluate the different resilience models, both moderation and mediation analyses were performed with the PROCESS-Macro for SPSS Version 27.0, Armonk, NY, USA: IBM Corp [41]. These analyses examine “how” (mediation) and “when” (moderation) an effect operates between an independent and an outcome variable [41]. The moderation and mediation analyses were performed separately for the outcome variables depression and anxiety symptoms.

For the moderation analysis (model 1 in PROCESS-Macro), subjective stress and resilience were included as independent predictors in the model in the first step. In a second step, the interaction term between the two predictors was added to the model. This procedure corresponds to a hierarchical regression and has been recommended in the literature for testing resilience models [42]. If the interaction term significantly predicts the outcome variable beyond the two independent predictors, it can be concluded that there is a significant moderation effect. Related to our hypotheses, only the main effects for resilience and stress would support a compensatory model while a significant interaction term between resilience and stress would confirm a protective model. In order to facilitate the interpretation of the main effects, both predictors were centered around the mean [41].

To test for the mediating effects of resilience between subjective stress burden and mental health symptoms, the indirect effect of resilience was calculated (see [41]; model 4 in PROCESS). The significance of the indirect effect can be tested using bootstrapping. If the 95% bias-corrected bootstrap confidence interval for the indirect effect (from 5000 bootstrap samples drawn) does not contain 0, it can be concluded that there is a significant indirect effect [41]. In addition, the total and direct effects were considered to determine the degree of mediation, and the proportion of the mediation was calculated by dividing the indirect effect by the total effect. For the moderation and mediation analyses, correlation coefficients and the corresponding 95% confidence intervals, as well as corresponding test statistics (*t*- and *p*-values), were presented. As recommended by Hayes (2012), heteroscedasticity-consistent standard errors for both analyses were computed using HC3 estimation.

## 3. Results

### 3.1. Associations between Sociodemographic/Occupational Characteristics and Resilience

The descriptive statistics for sociodemographic and occupational variables are shown in Table 1. Differences in resilience levels between different sociodemographic and occupational characteristics were tested with *t*-tests or ANOVAs (see Table 1). There were no significant associations between resilience and gender, living situation, occupation, work experience, job share and contact with the virus. The only significant mean differences were found for the variables age group and having a child. The ANOVA revealed a small effect for age, with higher age groups having higher resilience scores. The Games–Howell post hoc tests showed significant differences between the age groups that were not adjacent. Furthermore, resilience scores for people with at least one child were very slightly, but significantly higher than resilience scores for people who do not have a child. Regarding the multivariate analysis, the general linear model reached statistical significance (*F*(16, 1017) = 2.44, *p* = 0.001, η^2^*_p_* = 0.037). Again, only the variables age group and having children contributed significantly to the individual resilience scores, with an almost negligible effect for having children (Table 1).

### 3.2. Relationship between Resilience, Subjective Stress Burden in the Pandemic and Mental Health

The associations between the continuous variables of interest (resilience (RS-5), depression symptoms (PHQ-2), anxiety symptoms (GAD-2) and stress burden experienced) and age and gender as main demographic variables are shown in Table 2. Resilience was significantly negatively correlated with both screening measures for anxiety and depression symptoms as well as with subjective stress burden levels. Anxiety and depression symptoms and burden levels, however, had significant positive correlations with each other. Additionally, the total resilience score in the current sample was compared to the general German population [38], both measured with the RS-5. Welch’s *t*-test revealed a significantly higher level in the present study than in the German population before the pandemic (*M* = 27.20, *SD* = 5.20, *N = 4972*), *t*(1587) = 10.02, *p* < 0.001, *d* = 0.32).

#### 3.2.1. Moderating Effects of Resilience

The results of the moderation analyses using depression and anxiety symptoms as dependent variables are displayed in Table 3. Regarding depression symptoms, both the subjective stress burden due to the COVID-19 pandemic and the resilience scores significantly predicted the severity of depression symptoms. While subjective stress burden was positively associated with the outcome, individual resilience related negatively to symptoms of depression. Adding the interaction term did not significantly increase the explained variance of the model, and only the main effects remained statistically significant with no moderating effect of resilience. Examining the regression model for anxiety symptoms, similar results were found for the prediction of depression symptoms. There was also a positive relationship between experienced stress burden and anxiety symptoms, whereas higher resilience scores were associated with fewer anxiety symptoms. However, the significant interaction term in Step 2 of the model indicated a very small moderating effect of resilience while the main effects also remained significant.

**Figure 1:**
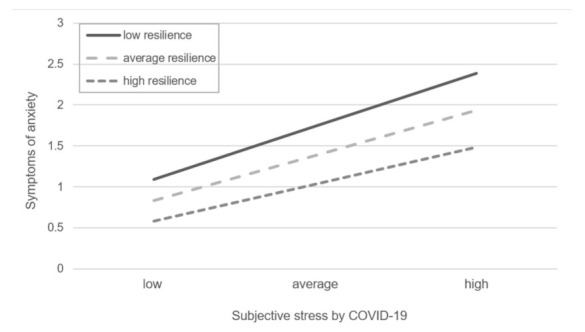
Simple slope analysis for low resilience (1 SD below the mean), average resilience and high resilience (1 SD above the mean).

To facilitate interpretation of the interaction effect, simple slope analyses were conducted for low (i.e., 1 SD below the mean), medium and high values (1 SD above the mean) of resilience. These are shown in Figure 1. In particular, the relationship between experienced stress and anxiety symptoms was weakened for high resilience scores, while it was more pronounced for low resilience scores. At the same time, independently of each other and indicated by the main effects, higher experienced stress burden and lower resilience levels led to stronger anxiety symptoms. These are shown in Table 4.

#### 3.2.2. Mediating Effects of Resilience

**Figure 2:**
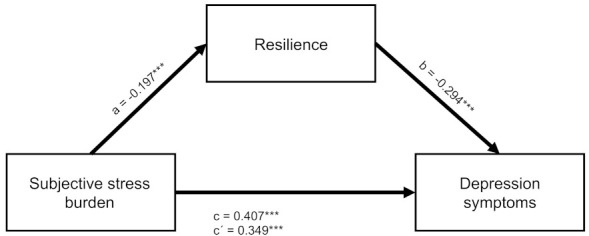
Partial mediation of the relationship between experienced stress and depression symptoms by resilience. Note: *N* = 1034. Standardized regression coefficients are shown. c = total effect. c’ = direct effect. a,b = indirect effect. *** *p* < 0.001.

Two mediation analyses were conducted to assess the mediating effects of resilience in the relationship between subjective stress burden and mental health symptoms. They were performed separately for depression and anxiety symptoms as target variables. The results of the mediation analysis for depression symptoms with the respective coefficients and the 95% bias-corrected bootstrap CI estimates are displayed in the left column of Table 5 and Figure 2.

A significant indirect effect of subjective stress burden on depression symptoms through resilience was found as there was no zero in the 95% bias-corrected bootstrap confidence interval for the indirect effect. However, the direct effect of subjective stress burden remained significant even after including resilience as mediator. Therefore, the relationship was only partially mediated with about 14% of the total effect explained by the mediation.

**Figure 3:**
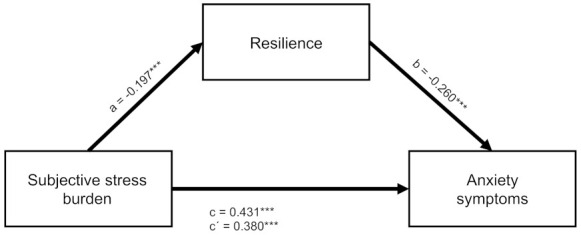
Partial mediation of the relationship between experienced stress and anxiety symptoms by resilience. Note: *N* = 1034. Standardized regression coefficients are shown. c = total effect. c’ = direct effect. a,b = indirect effect. *** *p* < 0.001.

The results of the mediation analysis for anxiety symptoms with the respective coefficients and the 95% bias-corrected bootstrap CI estimates are shown in the right column of Table 5 and Figure 3.

A significant indirect effect of subjective stress burden on anxiety symptoms through resilience was found as there was no zero in the 95% bias-corrected bootstrap confidence interval for the indirect effect. However, the direct effect of subjective stress burden remained significant even after including resilience as mediator. Therefore, the relationship was only partially mediated with about 12% of the total effect explained by the mediation.

## 4. Discussion

The present cross-sectional study focused on demographic and work-related correlates of resilience and the relationship of resilience with mental health symptoms in HCW in Germany during the COVID-19 pandemic. Being older and (with a small effect in the multifactorial analysis) having children were the only factors significantly associated with higher resilience scores. These results corroborate earlier findings that age in particular seems to be reliably associated with resilience scores [18,24,28,29]. Regarding the role of resilience in the relationship between COVID-19-related subjective stress and mental symptoms, the main effects of resilience provided evidence for a compensatory model, meaning that resilience and burden effects act independently on mental health. Additionally, an interaction between resilience scores and subjective stress also supported a protective model, at least for anxiety symptoms. Further analysis revealed that resilience partially mediated the relationship between stress and mental well-being. This result adds insights as to how resilience contributes to the mental well-being of HCW during the COVID-19 pandemic.

Resilience levels in the present survey were significantly higher than in a German population sample [38], possibly, but not only, due to differences in employment relationships and a very selected sample in our study. In contrast to Schmalbach et al. [38], all respondents in our sample were employed at the time of the survey, which has been associated with higher resilience levels in previous studies [43]. However, HCW, in particular, report even higher resilience levels than the general working population in other countries [44]. This suggests that differences in resilience levels extend beyond employment status and that additional factors such as education level [45], and also the timing of the survey, may play a role. With regard to the association between resilience and age, several studies have found links between higher resilience and older age in the health care sector [17,24,28,29] and in the general population [46,47], even though there are contrasting findings [48]. One possible explanation might be that resilience presumably develops in the context of stressful situations [49], and thus the accumulated knowledge of successfully dealing with challenges over a lifetime also influence the extent of an adult’s resilience [50]. This is consistent with the proposal that trait resilience is influenced by environmental events and life experiences [11]. Consistent with Chang et al. [28], the significant association in the multivariate analysis between age and resilience, while accounting for years of working experience, suggests that not only job experience, but also resources gained outside the workplace contribute to the individual resilience. Furthermore, socioeconomic status might influence the relationship, as older people usually have more financial security, which again is associated with higher resilience levels [51]. A possible selection effect in the sample could explain the findings as well. As high resilience in the healthcare sector is related to job satisfaction [52], fewer burnout symptoms [18,53] and fewer job change intentions [54,55], less resilient individuals may leave the healthcare system earlier than highly resilient individuals, thus explaining the positive correlation between age and resilience. In the present study, having children also had a small effect on resilience scores. Despite a lack of effects on having children in the literature, they could potentially increase resilience by eliciting positive emotions in their parents following the broaden-and-build theory [56]. However, it should be noted that in the multivariate analysis only very small effects were detected for having children, possibly due to confounding with the participants’ age. For this reason, the finding should be interpreted with caution. Taken together, all demographic and job-related predictors only seem to account only for a small amount of variance in resilience levels as measured by the RS-5 in HCW, a fact that has already been noted by other authors [57].

Another focus of the analysis was on different resilience effect models that could explain the relationship between COVID-19-related stress, resilience, and mental health outcomes. In the present study, both support for a compensatory model and a partial mediation was found, while the protective model only applied to anxiety symptoms as a dependent variable. Earlier research supports the notion that different resilience models can co-exist in a study [35]. These results, therefore, offer an insight into the ways by which resilience may work in a period of enhanced stress burden, such as a pandemic, among HCW. While individual resilience counteracted the negative effects of stress on mental symptoms independent of the subjective stress level, it also attenuated anxiety symptoms in particular for those with high COVID-19-related stress. Stronger moderating effects of resilience in the relationship between stress and anxiety symptoms have already been found in earlier studies [34,58]; however, Anyan and Hjemdal [35] only reported a moderating effect on depression symptoms, not on anxiety symptoms. As these studies differed regarding study population and questionnaires, further research is needed to corroborate the findings among HCW. A potential explanation may be that resilience is especially protective in highly stressful situations that evoke anxiety about something previously unknown (such as SARS-CoV-2). However, other aspects such as social support could play a stronger role in alleviating feelings of loneliness and depression caused by the increased stress.

While these results show “when” resilience operates, the mediation effects rather help to clarify “how” resilience works [cf. 34]. The partial mediation suggests that resilience explains part of the common variance between experienced stress and anxiety and depression symptoms, and thus possibly represents an element in the relationship that could be influenced by interventions. Nevertheless, only a small proportion, less than one-sixth, of the effect is mediated by resilience. This is consistent with previous studies that examined resilience as a possible mediator [59], including in the context of COVID-19 research [60]. Partial mediations were reported frequently in this context, though usually, they were not able to elucidate more than one-third of the total effect. Consequently, it seems logical that other resources besides resilience play a mediating role. Possible further factors that could be investigated are social support or a sense of coherence [60,61].

There are some limitations in our study to be considered. Firstly, for practical reasons, we used short versions of the original scales. While a low number of items may generally compromise the reliability of the measurements, the short scales used [PHQ-4: 35; RS-5: 36,37] have been widely applied in previous studies. Additionally, the internal consistencies in our sample were similar to the ones from the validating studies that reported acceptable [36] to good values [38]. We also measured the pandemic-related burden with a single item. This is likely insufficient to assess all aspects of the individual stress burden during the COVID-19 pandemic; however, in large samples, it can help to provide first insights into the research field and help to define directions for the future.

Secondly, causal inferences cannot be derived from the cross-sectional study design. Although the data suggest that resilience moderates and mediates the relationship between pandemic-related burdens and mental health symptoms, other interpretations are imaginable. For example, strong symptoms of depression or anxiety might lead participants to report lower resilience levels as a consequence. Therefore, longitudinal studies should corroborate the present findings.

Thirdly, a possible selection bias of the sample cannot be ruled out. Participants were recruited through an internal employee portal that addressed all HCW in a university hospital and via other general hospitals; however, most HCW are from one university hospital. However, we were able to provide estimates of representativeness, at least for the Bonn University Hospital, showing that the study sample is partially representative of HCW from three professional groups at the Bonn University Hospital, but presumably also for other German university hospitals [61] (especially for nurses and MTAs, and to a lesser extent for physicians).

The protective effects of resilience on mental health have significant implications for potential interventions for HCW that can promote individual resilience. Although there is still conceptual diversity regarding the definition of resilience [15], regardless of the theoretical discussion, it is crucial to improve the factors in HCW that help them adapt positively to adversity and lead to positive outcomes. Resilience training focusing on personal competence and associated resilient factors, and thus working in a resource-oriented manner, are a promising approach. These interventions could, for example, include improving self-efficacy, problem-solving skills, or the acceptance of one’s own emotions [62]. Further recommendations have also been proposed for building resilience during the COVID-19 pandemic [63]. The resilience models examined provide initial answers to whom the interventions should be directed. The compensatory resilience model found in the present work indicates that resilience-building interventions should be offered to all HCW, as risk factors and resilience act independently regardless of their exposure to the COVID-19 pandemic. The protective resilience model shown specifically for anxiety symptoms suggests a special focus on high-stressed HCW precisely to alleviate anxiety symptoms. Future research could test these associations with longitudinal designs and thus identify causal mechanisms and dynamic interactions between stressors and resilience. The associations between resilience and age indicate an additional focus on the resilience of younger-aged medical staff. In the wake of demographic change and the shortage of healthcare professionals, young people should be offered resilience-building measures as early as they start their careers in the healthcare sector, in order to avoid negative effects on their own health [64] and the healthcare system as a whole.

In light of substantially elevated prevalence rates of mental disorders during the COVID-19 pandemic, specifically in the health care settings, the current study provided important insights into the mechanisms of resilience for mental well-being and identified demographic correlates. We conclude that the development and evaluation of resilience-promoting measures in the health care system can be a powerful tool for the prevention of mental distress in times of a pandemic, especially for younger employees.

## Figures and Tables

**Table 1:** Descriptive statistics on demographics and occupation and differences in resilience levels (*N* = 1034).

	*n*	%	RS-5 ^a^ (M ± SD)	Univariate Test Statistics ^b^	Effect Size ^c^	*p*	Multivariate Test Statistics	Effect Size ^d^	*p*
*Gender*				*t*(625) = 0.82	0.05	0.413	*F*(11,017) = 0.35	0.000	0.553
Male	296	28.6	29.04 ± 4.28						
Female	738	71.4	28.78 ± 4.95						
*Age group*				*F*(4397) = 6.58	0.02	<**0.001**	*F*(41,017) = 3.32	0.013	**0.010**
18–30	192	18.6	27.88 ± 5.16						
31–40	227	22.0	28.41 ± 4.90						
41–50	191	18.5	28.57 ± 4.79						
51–60	333	32.2	29.52 ± 4.41						
>60	91	8.8	30.22 ± 4.15						
*Children*				*t*(966) = 3.83	0.24	<**0.001**	*F*(11,017) = 4.28	0.004	**0.039**
Yes	558	54.0	29.38 ± 4.50						
No	476	46.0	28.24 ± 5.00						
*Living situation*				*t*(377) = −1.23	0.09	0.221	*F*(11,017) = 0.11	0.000	0.739
Living alone	240	23.2	28.52 ± 4.97						
Not living alone	794	76.8	28.96 ± 4.70						
*Occupation*				*F*(4379) = 2.05	0.01	0.086	*F*(41,017) = 1.48	0.006	0.206
Physician	257	24.9	29.20 ± 4.30						
Nurse	330	31.9	28.54 ± 5.07						
MTA	90	8.7	27.88 ± 6.08						
PPE	154	14.9	29.43 ± 3.81						
Else	203	19.6	28.94 ± 4.76						
*Job Experience*				*F*(3180) = 0.98	0.00	0.403	*F*(31,017) = 0.27	0.001	0.850
<3 years	136	13.2	28.46 ± 4.24						
3–6 years	129	12.5	28.49 ± 4.93						
>6 years	713	69.0	29.02 ± 4.82						
unknown	56	5.4	28.57 ± 4.88						
*Fulltime employment*				*t*(707) = −0.27	0.02	0.788	*F*(11,017) = 0.78	0.001	0.378
Full time	701	67.8	28.83 ± 4.90						
Part time	333	32.2	28.91 ± 4.49						
*Contact with SARS-CoV-2*				*t*(879) = −0.73	0.05	0.463	*F*(11,017) = 1.35	0.001	0.246
yes	627	60.6	28.94 ± 4.80						
no	407	39.4	28.72 ± 4.71						

Note: ^a^ Resilience-Scale 5. ^b^ Results of two-tailed Welch’s *t*-test are reported for comparison between two groups and Welch-ANOVA for comparison between more than two groups. ^c^ Cohen’s ^d^ is reported for comparison between two groups and partial eta-squared for comparison between more than two groups. ^d^ Partial eta-squared is reported.

**Table 2:** Correlations between study variables.

Variable	M	SD	1	2	3	4	5	6
1. Gender ^a^								
2. Age ^b^			−0.11 ***					
3. Resilience (RS-5)	28.86	4.77	−0.02	0.15 ***				
4. Depression (PHQ-2)	1.54	1.37	0.06 *	−0.17 ***	−0.36 ***			
5. Anxiety (GAD-2)	1.41	1.43	0.07 *	−0.09 **	−0.34 ***	0.63 ***		
6. Subj. Stress Burden	2.13	0.96	0.07 *	0.00	−0.20 ***	0.41 ***	0.43 ***	

Note: *N* = 1034. ^a^ 0 = male and 1 = female. ^b^ Spearman’s Rho Coefficient is reported for the ordinal variable age. Pearson’s coefficient is reported for all other variables. *** *p* < 0.001. ** *p* < 0.01. * *p* < 0.05.

**Table 3:** Interaction effects of resilience and subjective stress burden on mental health.

	Model		R^2^	∆ R^2^	B	SE	ß	t	*p*
Depression Symptoms (PHQ-2)	1	(constant)			2.915	0.308		9.47	<0.001
		Burden	0.249	0.249	0.497	0.046	0.349	10.90	<0.001
		Resilience			−0.084	0.009	−0.294	−9.39	<0.001
	2	(constant)			1.533	0.039		39.63	<0.001
		Burden			0.501	0.046	0.351	10.83	<0.001
		Resilience	0.250	0.001	−0.083	0.009	−0.288	−9.26	<0.001
		Burden × Resilience			−0.010	0.009	−0.033	−1.06	0.291
Anxiety Symptoms (GAD-2)	1	(constant)			2.446	0.346		7.08	<0.001
		Burden	0.250	0.250	0.568	0.048	0.380	11.89	<0.001
		Resilience			−0.078	0.010	−0.260	−7.63	<0.001
	2	(constant)			1.386	0.040		34.32	<0.001
		Burden			0.575	0.048	0.384	11.95	<0.001
		Resilience	0.255	0.005	−0.074	0.010	−0.246	−7.50	<0.001
		Burden × Resilience			−0.021	0.010	−0.070	−2.25	0.025

**Table 4:** Effects of subjective stress burden on symptoms of anxiety at low, average and high levels of resilience.

Level of Moderator Variable	*B*	SE	*t*	*p*
Low resilience	0.68	0.07	9.53	<0.001
Average resilience	0.58	0.05	11.94	<0.001
High resilience	0.47	0.06	7.83	<0.001

Note: *N* = 1034. The categories are for low resilience (i.e., 1 SD below the mean), average resilience and high resilience (1 SD above the mean).

**Table 5:** Mediating effects of resilience in the relationship between subjective stress burden and mental health symptoms.

Effect	Depression Symptoms (PHQ-2)				Anxiety Symptoms (GAD-2)			
	B (SE)	ß	*p*-Value	Bias-Corrected Bootstrap 95% CI	B (SE)	ß	*p*-Value	Bias-Corrected Bootstrap 95% CI
***a***	−0.98 (0.15)	−0.20	<.001		−0.98 (0.15)	−0.20	<0.001	
***b***	−0.08 (0.01)	−0.29	<0.001		−0.08 (0.01)	−0.26	<0.001	
***c***	0.58 (0.04)	0.41	<0.001		0.64 (0.04)	0.43	<0.001	
c’	0.50 (0.04)	0.35	<0.001		0.57 (0.04)	0.38	<0.001	
***a*** × ***b***	0.08 (0.02)	0.06	<0.001	[0.05,0.12]	0.08 (0.02)	0.05	<0.001	[0.05,0.11]

Note: *N* = 1034. B = unstandardized path coefficient. ß = standardized path coefficient. SE = Standard Error. CI = confidence interval; a = effects of subjective stress burden on resilience; b = effects of resilience on depression and anxiety symptoms; c = total effect; c’ = direct effect; a × b= mediating effects of resilience in the relationship between subjective stress burden on anxiety and depression symptoms (i.e., the indirect effect).

## Data Availability

The authors cannot disclose individual-level data because of ethical and legal data protection restrictions and have included group-level data in the paper and tables. The dataset from this study is held securely in coded form at the Clinic for Psychosomatic Medicine (CPM) as part of the University Clinic and Medical Faculty of Bonn. While national data protection laws and ethical regulations, as well as cooperation agreements, prohibit the CPM from making the dataset publicly available, requests for access to anonymized or pooled data may be sent to the Data Access Committee of the CPM, Email: sabine.mueller@ukbonn.de.
